# Correction: ‘Inversion invasions: when the genetic basis of local adaptation is concentrated within inversions in the face of gene flow’ (2022), by Schaal *et al.*

**DOI:** 10.1098/rstb.2025.0102

**Published:** 2025-09-04

**Authors:** Kathleen Lotterhos

**Affiliations:** ^1^ Northeastern University, Boston, MA, USA

**Keywords:** structural rearrangements, low recombination, local adaptation, polygenic adaptation

*Phil. Trans. R. Soc. B* **377**: 20210200 (Published online 13 June 2022). (http://doi.org/10.1098/rstb.2021.0200)

An error was identified in equation (2.2). Relative fitness was normalized by dividing the fitness by the individual with the highest fitness within deme *p*.

The corrected equation is


$$w_{ip}=\exp\left(\frac{-{\left(z_{ip}-\Theta_{p}\right)}^{2}}{\omega_{p}^{2}}\right).$$

This change does not affect the results or conclusions of the paper.

